# Supplementary material for: Hot spot prediction in protein-protein interactions by an ensemble system
Source: BMC Syst Biol. 2018 Dec 31;12(Suppl 9):132. doi: 10.1186/s12918-018-0665-8 (PMC6311905; doi:10.1186/s12918-018-0665-8)
Supplement: Supplementary file 2 — All relevant MATLAB code. A simple Matlab implement of our predictor. (ZIP 19,742 kb) [file 12918_2018_665_MOESM2_ESM.zip › Additional file 2/Read ASA/ASA website/dbMPIKT/5C6TA.html]

 NetSurfP - Protein Surface Accessibility and Secondary Structure Predictions

  

|  |  |  |
| --- | --- | --- |
|  |  | NetSurfP - Protein Surface Accessibility and Secondary Structure Predictions  Technical University of Denmark |

  


---

```
# For publication of results, please cite:
# A generic method for assignment of reliability scores applied to solvent accessibility predictions.
# Bent Petersen, Thomas Nordahl Petersen, Pernille Andersen, Morten Nielsen and Claus Lundegaard
# BMC Structural Biology 2009, 9:51 doi:10.1186/1472-6807-9-51
#
# Column 1: Class assignment - B for buried or E for Exposed - Threshold: 25% exposure, but not based on RSA
# Column 2: Amino acid
# Column 3: Sequence name
# Column 4: Amino acid number
# Column 5: Relative Surface Accessibility - RSA
# Column 6: Absolute Surface Accessibility
# Column 7: Z-fit score for RSA prediction
# Column 8: Probability for Alpha-Helix
# Column 9: Probability for Beta-strand
# Column 10: Probability for Coil
E M  5C6TA                  1    0.785 157.179  -0.686   0.016   0.005   0.979
E E  5C6TA                  2    0.661 115.512  -0.387   0.052   0.084   0.864
E S  5C6TA                  3    0.445  52.142  -0.866   0.118   0.150   0.732
E R  5C6TA                  4    0.316  72.387   0.658   0.252   0.423   0.325
B I  5C6TA                  5    0.126  23.365  -0.756   0.391   0.466   0.143
B W  5C6TA                  6    0.094  22.511   1.046   0.391   0.466   0.143
B C  5C6TA                  7    0.032   4.507  -0.058   0.504   0.430   0.065
B L  5C6TA                  8    0.046   8.459   0.655   0.685   0.273   0.043
B V  5C6TA                  9    0.066  10.175   0.257   0.731   0.235   0.034
B V  5C6TA                 10    0.047   7.178   0.738   0.746   0.197   0.057
B C  5C6TA                 11    0.020   2.850   0.814   0.725   0.163   0.112
B V  5C6TA                 12    0.060   9.176  -0.081   0.701   0.107   0.192
B N  5C6TA                 13    0.189  27.626  -0.344   0.605   0.105   0.290
B L  5C6TA                 14    0.034   6.280   0.501   0.502   0.102   0.396
B C  5C6TA                 15    0.019   2.597   0.985   0.352   0.332   0.316
B I  5C6TA                 16    0.033   6.031   0.031   0.273   0.587   0.140
B V  5C6TA                 17    0.037   5.748  -0.035   0.157   0.773   0.069
B C  5C6TA                 18    0.023   3.243   0.486   0.085   0.775   0.140
B L  5C6TA                 19    0.040   7.306  -0.350   0.087   0.683   0.230
B G  5C6TA                 20    0.127  10.034  -0.903   0.022   0.359   0.619
B A  5C6TA                 21    0.192  21.180  -1.555   0.019   0.141   0.840
E A  5C6TA                 22    0.421  46.350  -1.897   0.018   0.047   0.935
E V  5C6TA                 23    0.293  44.973  -0.354   0.018   0.019   0.964
E S  5C6TA                 24    0.498  58.366  -0.598   0.018   0.019   0.964
E T  5C6TA                 25    0.661  91.736  -1.501   0.018   0.047   0.935
E K  5C6TA                 26    0.525 108.075   0.732   0.018   0.088   0.893
B Y  5C6TA                 27    0.136  29.085   1.254   0.004   0.085   0.910
B P  5C6TA                 28    0.206  29.274   0.345   0.005   0.262   0.733
B Y  5C6TA                 29    0.087  18.549  -0.074   0.003   0.718   0.279
B R  5C6TA                 30    0.141  32.197   0.297   0.001   0.900   0.099
B V  5C6TA                 31    0.048   7.408  -0.741   0.018   0.846   0.136
B C  5C6TA                 32    0.025   3.524   0.619   0.023   0.655   0.322
B S  5C6TA                 33    0.246  28.866  -0.704   0.005   0.262   0.733
B M  5C6TA                 34    0.130  26.073  -0.327   0.005   0.045   0.951
B A  5C6TA                 35    0.246  27.065  -1.211   0.018   0.019   0.964
E Q  5C6TA                 36    0.479  85.532  -0.869   0.018   0.019   0.964
E G  5C6TA                 37    0.394  30.992  -1.797   0.018   0.088   0.893
B T  5C6TA                 38    0.136  18.919  -1.082   0.021   0.279   0.699
E D  5C6TA                 39    0.263  37.956  -0.046   0.023   0.655   0.322
B L  5C6TA                 40    0.095  17.431   0.516   0.018   0.846   0.136
B I  5C6TA                 41    0.087  16.114   0.797   0.011   0.918   0.071
B R  5C6TA                 42    0.231  52.807   0.421   0.011   0.918   0.071
B F  5C6TA                 43    0.091  18.264   0.518   0.004   0.616   0.381
E E  5C6TA                 44    0.528  92.189   0.086   0.004   0.197   0.799
E R  5C6TA                 45    0.497 113.790  -0.053   0.018   0.088   0.893
E N  5C6TA                 46    0.621  90.929  -0.898   0.018   0.088   0.893
E I  5C6TA                 47    0.258  47.693   0.904   0.004   0.197   0.799
E V  5C6TA                 48    0.644  98.952   1.071   0.004   0.197   0.799
B C  5C6TA                 49    0.035   4.970  -1.415   0.005   0.045   0.951
E T  5C6TA                 50    0.504  69.960  -0.083   0.005   0.015   0.979
E S  5C6TA                 51    0.666  78.114   0.822   0.005   0.015   0.979
B M  5C6TA                 52    0.241  48.244  -0.005   0.018   0.019   0.964
E K  5C6TA                 53    0.603 124.119   0.436   0.018   0.047   0.935
E P  5C6TA                 54    0.478  67.885   0.079   0.018   0.047   0.935
E I  5C6TA                 55    0.596 110.205  -0.420   0.018   0.047   0.935
E N  5C6TA                 56    0.756 110.605  -0.677   0.018   0.019   0.964
E E  5C6TA                 57    0.484  84.572   1.054   0.018   0.088   0.893
E D  5C6TA                 58    0.526  75.782   0.803   0.019   0.141   0.840
B L  5C6TA                 59    0.149  27.190   1.289   0.066   0.296   0.638
E D  5C6TA                 60    0.336  48.389   0.273   0.074   0.484   0.442
B E  5C6TA                 61    0.117  20.527   0.098   0.079   0.592   0.329
B G  5C6TA                 62    0.042   3.321  -0.661   0.021   0.756   0.223
B I  5C6TA                 63    0.041   7.622   0.654   0.011   0.918   0.071
B M  5C6TA                 64    0.032   6.423   0.225   0.011   0.918   0.071
B V  5C6TA                 65    0.030   4.611   0.808   0.011   0.918   0.071
B V  5C6TA                 66    0.036   5.564   0.411   0.011   0.918   0.071
B Y  5C6TA                 67    0.101  21.520   0.601   0.011   0.918   0.071
B K  5C6TA                 68    0.232  47.764   0.412   0.021   0.756   0.223
E R  5C6TA                 69    0.503 115.233   0.741   0.066   0.296   0.638
E N  5C6TA                 70    0.437  63.948  -0.474   0.064   0.216   0.721
B I  5C6TA                 71    0.121  22.404   0.320   0.064   0.216   0.721
E V  5C6TA                 72    0.300  46.171   0.235   0.022   0.359   0.619
B A  5C6TA                 73    0.265  29.214  -0.293   0.021   0.451   0.528
B H  5C6TA                 74    0.160  29.104   0.700   0.021   0.756   0.223
E T  5C6TA                 75    0.338  46.867   0.537   0.018   0.846   0.136
B F  5C6TA                 76    0.091  18.244   0.659   0.018   0.846   0.136
E K  5C6TA                 77    0.425  87.423   1.194   0.018   0.846   0.136
B V  5C6TA                 78    0.045   6.993   0.194   0.011   0.918   0.071
B R  5C6TA                 79    0.234  53.540   0.588   0.001   0.900   0.099
B V  5C6TA                 80    0.165  25.330   0.808   0.001   0.900   0.099
B Y  5C6TA                 81    0.099  21.220   0.346   0.001   0.900   0.099
B Q  5C6TA                 82    0.241  43.114   0.875   0.001   0.900   0.099
B K  5C6TA                 83    0.192  39.556   0.222   0.001   0.900   0.099
E V  5C6TA                 84    0.278  42.729  -0.148   0.001   0.900   0.099
B L  5C6TA                 85    0.085  15.490   0.562   0.001   0.959   0.040
B T  5C6TA                 86    0.219  30.389  -0.040   0.001   0.959   0.040
B F  5C6TA                 87    0.172  34.520  -0.231   0.001   0.959   0.040
B R  5C6TA                 88    0.241  55.189   0.394   0.001   0.959   0.040
B R  5C6TA                 89    0.174  39.777   0.753   0.001   0.900   0.099
B S  5C6TA                 90    0.188  22.010  -0.538   0.001   0.900   0.099
B Y  5C6TA                 91    0.174  37.227   0.042   0.002   0.816   0.182
E A  5C6TA                 92    0.336  37.038  -0.697   0.023   0.655   0.322
B Y  5C6TA                 93    0.256  54.750  -1.999   0.023   0.655   0.322
B H  5C6TA                 94    0.226  41.146  -0.029   0.021   0.756   0.223
B R  5C6TA                 95    0.191  43.808  -0.264   0.021   0.756   0.223
B T  5C6TA                 96    0.220  30.472   0.184   0.018   0.846   0.136
B T  5C6TA                 97    0.257  35.646   0.032   0.021   0.756   0.223
B Y  5C6TA                 98    0.175  37.312   0.265   0.021   0.756   0.223
B L  5C6TA                 99    0.098  17.889  -1.005   0.021   0.756   0.223
B L  5C6TA                100    0.158  28.966   0.105   0.022   0.552   0.426
E G  5C6TA                101    0.343  27.002  -0.995   0.022   0.359   0.619
E S  5C6TA                102    0.443  51.873   0.831   0.022   0.359   0.619
E N  5C6TA                103    0.333  48.678  -0.661   0.022   0.552   0.426
E T  5C6TA                104    0.402  55.799  -0.329   0.022   0.552   0.426
E E  5C6TA                105    0.417  72.850  -0.884   0.022   0.552   0.426
E Y  5C6TA                106    0.411  87.809  -0.259   0.021   0.451   0.528
B V  5C6TA                107    0.238  36.581   0.360   0.021   0.451   0.528
E A  5C6TA                108    0.326  35.958  -0.681   0.022   0.359   0.619
B P  5C6TA                109    0.078  11.011   0.264   0.019   0.141   0.840
E P  5C6TA                110    0.298  42.229   0.227   0.053   0.043   0.903
B M  5C6TA                111    0.249  49.765  -0.873   0.386   0.097   0.517
B W  5C6TA                112    0.325  78.114  -0.597   0.428   0.171   0.402
B E  5C6TA                113    0.141  24.703  -0.731   0.428   0.171   0.402
B I  5C6TA                114    0.072  13.357   0.209   0.453   0.248   0.299
E H  5C6TA                115    0.411  74.725   0.267   0.321   0.252   0.427
E H  5C6TA                116    0.345  62.701   0.937   0.321   0.252   0.427
B I  5C6TA                117    0.097  17.908   0.050   0.216   0.235   0.548
B N  5C6TA                118    0.265  38.855  -0.199   0.191   0.086   0.723
E S  5C6TA                119    0.462  54.123  -0.497   0.113   0.087   0.800
E H  5C6TA                120    0.428  77.926   0.290   0.118   0.150   0.732
B S  5C6TA                121    0.207  24.225  -0.438   0.069   0.386   0.545
E Q  5C6TA                122    0.352  62.796   0.785   0.023   0.655   0.322
B C  5C6TA                123    0.031   4.366  -0.633   0.023   0.655   0.322
B Y  5C6TA                124    0.202  43.232   0.305   0.023   0.655   0.322
B S  5C6TA                125    0.123  14.416  -1.436   0.022   0.552   0.426
B S  5C6TA                126    0.129  15.060  -0.364   0.087   0.683   0.230
B Y  5C6TA                127    0.085  18.271  -0.481   0.018   0.846   0.136
E S  5C6TA                128    0.326  38.207   1.181   0.006   0.962   0.032
B R  5C6TA                129    0.151  34.579   1.123   0.006   0.962   0.032
B V  5C6TA                130    0.239  36.688   0.457   0.006   0.962   0.032
E I  5C6TA                131    0.333  61.661   0.752   0.002   0.816   0.182
E A  5C6TA                132    0.585  64.511  -1.629   0.004   0.197   0.799
E G  5C6TA                133    0.606  47.676  -0.593   0.004   0.085   0.910
E T  5C6TA                134    0.295  40.930   0.231   0.002   0.816   0.182
E V  5C6TA                135    0.320  49.123   0.791   0.001   0.959   0.040
B F  5C6TA                136    0.153  30.607   1.337   0.001   0.959   0.040
B V  5C6TA                137    0.155  23.747   0.979   0.001   0.959   0.040
B A  5C6TA                138    0.048   5.312   0.632   0.001   0.959   0.040
B Y  5C6TA                139    0.127  27.119   0.566   0.001   0.900   0.099
B H  5C6TA                140    0.112  20.427  -0.049   0.003   0.718   0.279
E R  5C6TA                141    0.382  87.547   0.242   0.018   0.088   0.893
E D  5C6TA                142    0.472  68.030  -0.865   0.018   0.019   0.964
E S  5C6TA                143    0.695  81.501  -0.892   0.018   0.019   0.964
E Y  5C6TA                144    0.412  88.087  -0.772   0.018   0.047   0.935
E E  5C6TA                145    0.584 102.077   0.427   0.021   0.279   0.699
E N  5C6TA                146    0.460  67.300   0.051   0.004   0.420   0.576
E K  5C6TA                147    0.367  75.574   1.177   0.003   0.718   0.279
E T  5C6TA                148    0.343  47.574   0.286   0.002   0.816   0.182
B M  5C6TA                149    0.081  16.148   1.056   0.002   0.816   0.182
E Q  5C6TA                150    0.269  48.115   0.650   0.002   0.816   0.182
B L  5C6TA                151    0.031   5.658  -0.265   0.002   0.816   0.182
B M  5C6TA                152    0.228  45.663   1.025   0.002   0.816   0.182
B P  5C6TA                153    0.172  24.350  -1.409   0.004   0.616   0.381
B D  5C6TA                154    0.267  38.417  -0.958   0.021   0.279   0.699
E D  5C6TA                155    0.610  87.944  -0.431   0.018   0.088   0.893
E Y  5C6TA                156    0.375  80.095  -1.975   0.018   0.047   0.935
E S  5C6TA                157    0.475  55.717  -1.680   0.018   0.047   0.935
E N  5C6TA                158    0.322  47.170  -1.628   0.018   0.047   0.935
E T  5C6TA                159    0.643  89.198  -1.274   0.018   0.047   0.935
E H  5C6TA                160    0.539  98.099  -0.741   0.019   0.141   0.840
B S  5C6TA                161    0.123  14.416  -0.826   0.005   0.262   0.733
E T  5C6TA                162    0.373  51.749   0.033   0.004   0.616   0.381
B R  5C6TA                163    0.160  36.732  -0.288   0.002   0.816   0.182
B Y  5C6TA                164    0.152  32.418   0.205   0.002   0.816   0.182
B V  5C6TA                165    0.153  23.562  -0.137   0.002   0.816   0.182
B T  5C6TA                166    0.206  28.628  -0.481   0.003   0.718   0.279
B V  5C6TA                167    0.153  23.516   0.464   0.022   0.359   0.619
E K  5C6TA                168    0.525 108.054   0.171   0.019   0.141   0.840
E D  5C6TA                169    0.384  55.378  -0.412   0.020   0.205   0.775
E Q  5C6TA                170    0.327  58.474  -0.449   0.021   0.451   0.528
B W  5C6TA                171    0.226  54.305  -0.220   0.021   0.451   0.528
B H  5C6TA                172    0.211  38.363  -0.104   0.021   0.451   0.528
E S  5C6TA                173    0.512  60.018  -0.245   0.021   0.279   0.699
E R  5C6TA                174    0.338  77.287  -1.269   0.019   0.141   0.840
E G  5C6TA                175    0.318  25.027  -1.275   0.005   0.045   0.951
B S  5C6TA                176    0.196  23.030  -0.908   0.004   0.085   0.910
B T  5C6TA                177    0.117  16.242  -0.533   0.005   0.336   0.660
B N  5C6TA                178    0.088  12.912  -1.065   0.003   0.718   0.279
B L  5C6TA                179    0.105  19.171  -0.272   0.002   0.816   0.182
B T  5C6TA                180    0.086  11.873   0.085   0.011   0.918   0.071
B R  5C6TA                181    0.183  41.930   0.062   0.002   0.816   0.182
B E  5C6TA                182    0.194  33.962  -0.305   0.004   0.616   0.381
E T  5C6TA                183    0.301  41.818  -0.274   0.004   0.514   0.481
B S  5C6TA                184    0.076   8.931  -0.520   0.004   0.514   0.481
B N  5C6TA                185    0.161  23.512  -0.791   0.004   0.616   0.381
B L  5C6TA                186    0.068  12.432  -0.331   0.004   0.514   0.481
B N  5C6TA                187    0.244  35.766   0.019   0.004   0.616   0.381
B C  5C6TA                188    0.037   5.181   0.117   0.001   0.900   0.099
B M  5C6TA                189    0.190  38.119   0.626   0.001   0.959   0.040
B V  5C6TA                190    0.084  12.849   0.866   0.001   0.959   0.040
B T  5C6TA                191    0.258  35.798   1.010   0.001   0.959   0.040
B I  5C6TA                192    0.294  54.353   0.192   0.011   0.918   0.071
B T  5C6TA                193    0.146  20.264   0.626   0.018   0.846   0.136
E T  5C6TA                194    0.348  48.309   0.270   0.018   0.846   0.136
B A  5C6TA                195    0.131  14.403  -0.898   0.023   0.655   0.322
E R  5C6TA                196    0.405  92.653  -0.115   0.021   0.451   0.528
B S  5C6TA                197    0.250  29.335  -0.931   0.020   0.205   0.775
E K  5C6TA                198    0.506 103.981   0.197   0.018   0.088   0.893
E Y  5C6TA                199    0.356  76.163   0.068   0.018   0.047   0.935
E P  5C6TA                200    0.274  38.824  -0.556   0.018   0.047   0.935
B Y  5C6TA                201    0.147  31.371   0.237   0.052   0.084   0.864
E H  5C6TA                202    0.429  78.053  -0.100   0.118   0.150   0.732
B F  5C6TA                203    0.162  32.473   0.192   0.135   0.317   0.548
B F  5C6TA                204    0.058  11.580   0.555   0.074   0.484   0.442
B A  5C6TA                205    0.070   7.670  -0.594   0.074   0.484   0.442
B T  5C6TA                206    0.106  14.688  -0.075   0.069   0.386   0.545
B S  5C6TA                207    0.176  20.627  -0.658   0.064   0.216   0.721
B T  5C6TA                208    0.257  35.660  -0.861   0.052   0.084   0.864
E G  5C6TA                209    0.282  22.154  -1.064   0.018   0.047   0.935
E D  5C6TA                210    0.318  45.867  -0.359   0.018   0.088   0.893
B V  5C6TA                211    0.161  24.807   0.341   0.003   0.718   0.279
B V  5C6TA                212    0.066  10.144   0.403   0.011   0.918   0.071
B D  5C6TA                213    0.188  27.019  -0.297   0.011   0.918   0.071
B I  5C6TA                214    0.042   7.770  -0.401   0.018   0.846   0.136
B S  5C6TA                215    0.054   6.387   0.416   0.004   0.514   0.481
B P  5C6TA                216    0.094  13.310  -0.480   0.005   0.262   0.733
B F  5C6TA                217    0.065  13.126  -1.258   0.021   0.279   0.699
B Y  5C6TA                218    0.165  35.175  -0.218   0.021   0.279   0.699
E N  5C6TA                219    0.387  56.671  -0.956   0.018   0.088   0.893
E G  5C6TA                220    0.487  38.351  -1.720   0.018   0.019   0.964
E T  5C6TA                221    0.669  92.763  -1.936   0.018   0.019   0.964
E N  5C6TA                222    0.564  82.526  -1.562   0.018   0.019   0.964
E R  5C6TA                223    0.596 136.415  -0.007   0.018   0.019   0.964
E N  5C6TA                224    0.557  81.486  -0.692   0.018   0.047   0.935
E A  5C6TA                225    0.475  52.389  -0.594   0.018   0.047   0.935
E S  5C6TA                226    0.603  70.707   0.031   0.019   0.141   0.840
E Y  5C6TA                227    0.427  91.186   0.087   0.020   0.205   0.775
B F  5C6TA                228    0.225  45.157  -1.070   0.020   0.205   0.775
E G  5C6TA                229    0.498  39.216  -1.304   0.019   0.141   0.840
E E  5C6TA                230    0.336  58.717  -0.973   0.019   0.141   0.840
E N  5C6TA                231    0.366  53.626  -0.311   0.018   0.047   0.935
E A  5C6TA                232    0.313  34.471  -1.217   0.018   0.047   0.935
E D  5C6TA                233    0.343  49.441  -1.457   0.019   0.141   0.840
B K  5C6TA                234    0.228  46.858   0.943   0.022   0.552   0.426
B F  5C6TA                235    0.039   7.867  -0.226   0.018   0.846   0.136
B F  5C6TA                236    0.291  58.323   1.342   0.006   0.962   0.032
B I  5C6TA                237    0.167  30.969  -0.049   0.011   0.918   0.071
B F  5C6TA                238    0.170  34.039   0.775   0.021   0.756   0.223
E P  5C6TA                239    0.608  86.275  -0.021   0.005   0.262   0.733
E N  5C6TA                240    0.621  90.973  -0.284   0.004   0.085   0.910
B Y  5C6TA                241    0.119  25.388   0.604   0.005   0.336   0.660
E T  5C6TA                242    0.377  52.345   0.789   0.002   0.816   0.182
B I  5C6TA                243    0.211  39.035   0.686   0.011   0.918   0.071
B V  5C6TA                244    0.181  27.804   1.077   0.018   0.846   0.136
E S  5C6TA                245    0.359  42.040  -0.260   0.021   0.756   0.223
B D  5C6TA                246    0.245  35.362   0.051   0.021   0.451   0.528
E F  5C6TA                247    0.485  97.259  -1.510   0.056   0.142   0.802
E G  5C6TA                248    0.546  42.947  -1.585   0.053   0.043   0.903
E R  5C6TA                249    0.492 112.645  -0.910   0.052   0.084   0.864
E P  5C6TA                250    0.490  69.474  -1.404   0.018   0.088   0.893
E N  5C6TA                251    0.554  81.164  -0.608   0.018   0.088   0.893
E S  5C6TA                252    0.299  35.090  -0.231   0.018   0.047   0.935
E A  5C6TA                253    0.555  61.183  -0.612   0.018   0.047   0.935
E L  5C6TA                254    0.553 101.199   0.227   0.053   0.043   0.903
E E  5C6TA                255    0.538  94.076  -0.046   0.056   0.142   0.802
E T  5C6TA                256    0.416  57.769  -0.426   0.066   0.296   0.638
E H  5C6TA                257    0.262  47.658   0.580   0.022   0.359   0.619
E R  5C6TA                258    0.413  94.691   0.172   0.069   0.386   0.545
E L  5C6TA                259    0.290  53.081   0.049   0.079   0.592   0.329
B V  5C6TA                260    0.191  29.403   0.713   0.023   0.655   0.322
B A  5C6TA                261    0.096  10.612  -0.324   0.023   0.655   0.322
B F  5C6TA                262    0.076  15.213  -0.432   0.023   0.655   0.322
B L  5C6TA                263    0.082  14.978  -0.416   0.023   0.655   0.322
E E  5C6TA                264    0.419  73.269   0.237   0.022   0.552   0.426
E R  5C6TA                265    0.350  80.242   1.239   0.018   0.088   0.893
E A  5C6TA                266    0.485  53.458  -0.566   0.005   0.045   0.951
E D  5C6TA                267    0.515  74.197  -0.120   0.004   0.197   0.799
B S  5C6TA                268    0.089  10.489   0.452   0.002   0.816   0.182
B V  5C6TA                269    0.138  21.272   0.126   0.011   0.918   0.071
B I  5C6TA                270    0.081  14.911   1.011   0.001   0.959   0.040
B S  5C6TA                271    0.069   8.052  -1.141   0.011   0.918   0.071
B W  5C6TA                272    0.087  20.923   0.740   0.018   0.846   0.136
E D  5C6TA                273    0.463  66.747   1.129   0.018   0.846   0.136
B I  5C6TA                274    0.164  30.377   0.285   0.021   0.756   0.223
E Q  5C6TA                275    0.426  76.084   0.802   0.023   0.655   0.322
E D  5C6TA                276    0.524  75.508   0.739   0.020   0.205   0.775
E E  5C6TA                277    0.631 110.236   1.003   0.018   0.047   0.935
E K  5C6TA                278    0.605 124.469   0.759   0.019   0.141   0.840
E N  5C6TA                279    0.568  83.111   0.462   0.066   0.296   0.638
E V  5C6TA                280    0.286  43.897   0.150   0.069   0.386   0.545
E T  5C6TA                281    0.371  51.402   0.908   0.079   0.592   0.329
B C  5C6TA                282    0.055   7.764   0.688   0.079   0.592   0.329
E Q  5C6TA                283    0.411  73.405   0.883   0.169   0.612   0.219
B L  5C6TA                284    0.138  25.195   0.772   0.373   0.401   0.226
E T  5C6TA                285    0.341  47.283   0.817   0.478   0.309   0.214
B F  5C6TA                286    0.247  49.613   0.988   0.594   0.285   0.121
B W  5C6TA                287    0.099  23.737  -0.033   0.578   0.229   0.194
E E  5C6TA                288    0.334  58.315   1.214   0.538   0.173   0.289
E A  5C6TA                289    0.447  49.303   0.268   0.502   0.102   0.396
B S  5C6TA                290    0.080   9.317   0.693   0.660   0.049   0.291
E E  5C6TA                291    0.394  68.814   0.144   0.751   0.050   0.199
E R  5C6TA                292    0.397  91.005   0.384   0.751   0.050   0.199
B T  5C6TA                293    0.120  16.658  -0.744   0.751   0.050   0.199
B I  5C6TA                294    0.072  13.264   0.202   0.779   0.100   0.120
E R  5C6TA                295    0.309  70.853   0.704   0.779   0.100   0.120
B S  5C6TA                296    0.249  29.230  -0.214   0.605   0.105   0.290
E E  5C6TA                297    0.414  72.396   0.068   0.455   0.046   0.498
E A  5C6TA                298    0.291  32.101  -0.188   0.268   0.043   0.689
E E  5C6TA                299    0.648 113.188   0.028   0.058   0.017   0.925
E D  5C6TA                300    0.549  79.154  -0.599   0.053   0.043   0.903
B S  5C6TA                301    0.246  28.808  -0.363   0.064   0.216   0.721
B Y  5C6TA                302    0.114  24.362   0.638   0.079   0.592   0.329
B H  5C6TA                303    0.233  42.437   0.851   0.085   0.775   0.140
B F  5C6TA                304    0.080  16.016   0.171   0.072   0.855   0.073
B S  5C6TA                305    0.162  18.998   0.060   0.085   0.775   0.140
B S  5C6TA                306    0.091  10.677  -0.145   0.169   0.612   0.219
E A  5C6TA                307    0.509  56.070   0.117   0.125   0.227   0.648
E K  5C6TA                308    0.468  96.165   0.451   0.125   0.227   0.648
B M  5C6TA                309    0.157  31.316  -0.143   0.069   0.386   0.545
B T  5C6TA                310    0.243  33.732  -0.332   0.087   0.683   0.230
B A  5C6TA                311    0.099  10.866  -0.580   0.018   0.846   0.136
B T  5C6TA                312    0.212  29.418  -0.446   0.018   0.846   0.136
B F  5C6TA                313    0.122  24.405  -0.195   0.018   0.846   0.136
B L  5C6TA                314    0.200  36.565  -0.542   0.023   0.655   0.322
B S  5C6TA                315    0.134  15.717  -0.288   0.022   0.359   0.619
E K  5C6TA                316    0.560 115.130  -0.074   0.019   0.141   0.840
E K  5C6TA                317    0.452  92.997  -0.148   0.018   0.088   0.893
E Q  5C6TA                318    0.487  86.978  -0.491   0.019   0.141   0.840
E E  5C6TA                319    0.580 101.274  -0.550   0.066   0.296   0.638
E V  5C6TA                320    0.300  46.187  -0.377   0.066   0.296   0.638
E N  5C6TA                321    0.503  73.610  -0.973   0.056   0.142   0.802
E M  5C6TA                322    0.338  67.674  -1.068   0.113   0.087   0.800
E S  5C6TA                323    0.381  44.712  -1.615   0.113   0.043   0.844
E D  5C6TA                324    0.562  80.984  -0.344   0.113   0.043   0.844
E S  5C6TA                325    0.473  55.424  -0.115   0.268   0.043   0.689
E A  5C6TA                326    0.486  53.590   0.214   0.278   0.093   0.628
B L  5C6TA                327    0.107  19.555   0.911   0.307   0.165   0.527
E D  5C6TA                328    0.585  84.299   1.347   0.278   0.093   0.628
B C  5C6TA                329    0.080  11.162  -0.053   0.502   0.102   0.396
B V  5C6TA                330    0.103  15.770   0.502   0.455   0.046   0.498
E R  5C6TA                331    0.376  86.012   0.777   0.717   0.014   0.269
E D  5C6TA                332    0.683  98.449   1.264   0.923   0.002   0.076
E E  5C6TA                333    0.417  72.920   0.894   0.970   0.001   0.030
B A  5C6TA                334    0.032   3.504  -0.455   0.988   0.000   0.012
E I  5C6TA                335    0.408  75.443   1.750   0.988   0.000   0.012
E N  5C6TA                336    0.554  81.164   1.867   0.988   0.000   0.012
E K  5C6TA                337    0.253  52.145   1.430   0.988   0.000   0.012
B L  5C6TA                338    0.033   6.006   0.646   0.988   0.000   0.012
E Q  5C6TA                339    0.412  73.673   1.405   0.988   0.000   0.012
E Q  5C6TA                340    0.576 102.838   1.712   0.988   0.000   0.012
B I  5C6TA                341    0.226  41.884   1.234   0.988   0.000   0.012
B F  5C6TA                342    0.093  18.585   0.483   0.970   0.001   0.030
E N  5C6TA                343    0.480  70.301   1.149   0.970   0.001   0.030
E T  5C6TA                344    0.588  81.583   1.057   0.923   0.002   0.076
E S  5C6TA                345    0.490  57.440   1.383   0.858   0.002   0.139
B Y  5C6TA                346    0.124  26.392   0.295   0.600   0.003   0.397
E N  5C6TA                347    0.482  70.579   0.269   0.430   0.016   0.555
E Q  5C6TA                348    0.656 117.126   0.547   0.268   0.043   0.689
E T  5C6TA                349    0.344  47.782   0.118   0.199   0.152   0.649
B Y  5C6TA                350    0.262  55.947   0.044   0.135   0.317   0.548
E E  5C6TA                351    0.265  46.243   0.414   0.066   0.296   0.638
E K  5C6TA                352    0.541 111.243   0.182   0.064   0.216   0.721
E Y  5C6TA                353    0.559 119.394  -1.394   0.018   0.088   0.893
E G  5C6TA                354    0.561  44.111  -1.087   0.018   0.047   0.935
E N  5C6TA                355    0.586  85.790   0.814   0.004   0.138   0.858
B V  5C6TA                356    0.159  24.438   0.559   0.003   0.718   0.279
E S  5C6TA                357    0.337  39.532   0.854   0.011   0.918   0.071
B V  5C6TA                358    0.202  31.032   1.480   0.001   0.959   0.040
B F  5C6TA                359    0.098  19.649   0.918   0.001   0.959   0.040
E E  5C6TA                360    0.280  48.951   0.940   0.001   0.959   0.040
B T  5C6TA                361    0.089  12.400  -0.784   0.002   0.816   0.182
E T  5C6TA                362    0.432  59.946  -0.356   0.005   0.262   0.733
E G  5C6TA                363    0.281  22.115  -1.241   0.018   0.047   0.935
B G  5C6TA                364    0.183  14.434  -0.964   0.018   0.088   0.893
B L  5C6TA                365    0.055  10.125   0.633   0.018   0.846   0.136
B V  5C6TA                366    0.063   9.698   0.986   0.006   0.962   0.032
B V  5C6TA                367    0.042   6.471   1.130   0.006   0.962   0.032
B F  5C6TA                368    0.038   7.647  -0.037   0.006   0.962   0.032
B W  5C6TA                369    0.068  16.450   0.038   0.011   0.918   0.071
B Q  5C6TA                370    0.173  30.934   0.803   0.023   0.655   0.322
B G  5C6TA                371    0.254  19.974  -0.812   0.064   0.216   0.721
B I  5C6TA                372    0.144  26.585   0.295   0.113   0.043   0.844
E K  5C6TA                373    0.281  57.884   1.076   0.058   0.017   0.925
E Q  5C6TA                374    0.404  72.190   0.254   0.430   0.016   0.555
E K  5C6TA                375    0.501 103.138   0.334   0.600   0.003   0.397
E S  5C6TA                376    0.347  40.622   0.295   0.600   0.003   0.397
B L  5C6TA                377    0.082  15.033  -0.127   0.923   0.002   0.076
E V  5C6TA                378    0.345  53.073   0.927   0.923   0.002   0.076
E E  5C6TA                379    0.482  84.205   1.503   0.970   0.001   0.030
B L  5C6TA                380    0.065  11.956   0.942   0.923   0.002   0.076
E E  5C6TA                381    0.333  58.228   0.574   0.923   0.002   0.076
E R  5C6TA                382    0.471 107.951   0.545   0.923   0.002   0.076
B L  5C6TA                383    0.241  44.109  -0.412   0.782   0.003   0.216
E A  5C6TA                384    0.335  36.884  -0.739   0.600   0.003   0.397
E N  5C6TA                385    0.668  97.854  -0.413   0.321   0.003   0.675
E R  5C6TA                386    0.464 106.279  -0.353   0.181   0.016   0.803
E S  5C6TA                387    0.519  60.862  -0.719   0.181   0.016   0.803
E S  5C6TA                388    0.445  52.166  -0.727   0.113   0.043   0.844
E L  5C6TA                389    0.512  93.747  -0.451   0.053   0.043   0.903
E N  5C6TA                390    0.599  87.752  -1.240   0.053   0.043   0.903
E L  5C6TA                391    0.440  80.546  -1.079   0.058   0.017   0.925
E T  5C6TA                392    0.516  71.569  -1.027   0.058   0.017   0.925
E H  5C6TA                393    0.468  85.056  -0.721   0.058   0.017   0.925
E N  5C6TA                394    0.534  78.119  -0.932   0.053   0.043   0.903
E S  5C6TA                395    0.568  66.605  -1.007   0.053   0.043   0.903
E T  5C6TA                396    0.485  67.200  -0.937   0.053   0.043   0.903
E K  5C6TA                397    0.544 111.963  -0.294   0.052   0.084   0.864
E S  5C6TA                398    0.454  53.232  -0.718   0.052   0.084   0.864
E S  5C6TA                399    0.516  60.452  -0.401   0.052   0.084   0.864
E T  5C6TA                400    0.486  67.422  -0.963   0.053   0.043   0.903
E D  5C6TA                401    0.536  77.252  -0.800   0.053   0.043   0.903
E G  5C6TA                402    0.570  44.843  -1.547   0.113   0.043   0.844
E N  5C6TA                403    0.557  81.603  -1.019   0.113   0.043   0.844
E N  5C6TA                404    0.536  78.456  -0.454   0.113   0.087   0.800
E A  5C6TA                405    0.320  35.264  -1.441   0.056   0.142   0.802
E T  5C6TA                406    0.414  57.380  -0.182   0.056   0.142   0.802
E H  5C6TA                407    0.515  93.660  -0.618   0.064   0.216   0.721
E L  5C6TA                408    0.451  82.596  -0.862   0.064   0.216   0.721
E S  5C6TA                409    0.417  48.872  -0.414   0.069   0.386   0.545
E N  5C6TA                410    0.433  63.377  -0.101   0.074   0.484   0.442
E M  5C6TA                411    0.323  64.552  -0.365   0.074   0.484   0.442
E E  5C6TA                412    0.515  89.953  -0.394   0.074   0.484   0.442
E S  5C6TA                413    0.314  36.859  -0.571   0.069   0.386   0.545
E V  5C6TA                414    0.386  59.359  -1.224   0.125   0.227   0.648
E H  5C6TA                415    0.463  84.220  -0.847   0.113   0.043   0.844
B N  5C6TA                416    0.255  37.347  -0.109   0.268   0.043   0.689
B L  5C6TA                417    0.080  14.630   0.243   0.605   0.105   0.290
B V  5C6TA                418    0.188  28.926   0.176   0.662   0.225   0.113
B Y  5C6TA                419    0.120  25.537   0.737   0.662   0.225   0.113
B A  5C6TA                420    0.056   6.171   0.041   0.725   0.163   0.112
B Q  5C6TA                421    0.107  19.057   0.362   0.779   0.100   0.120
B L  5C6TA                422    0.037   6.701   0.081   0.870   0.077   0.053
B Q  5C6TA                423    0.122  21.861   0.790   0.911   0.033   0.057
B F  5C6TA                424    0.141  28.319   0.184   0.911   0.033   0.057
B T  5C6TA                425    0.042   5.867   0.053   0.879   0.010   0.111
B Y  5C6TA                426    0.100  21.413   0.583   0.923   0.002   0.076
E D  5C6TA                427    0.377  54.398   0.622   0.970   0.001   0.030
B T  5C6TA                428    0.255  35.299   1.395   0.970   0.001   0.030
B L  5C6TA                429    0.029   5.292   0.336   0.970   0.001   0.030
B R  5C6TA                430    0.243  55.555   1.177   0.988   0.000   0.012
E G  5C6TA                431    0.584  45.953   1.555   0.970   0.001   0.030
B Y  5C6TA                432    0.193  41.180   1.023   0.970   0.001   0.030
B I  5C6TA                433    0.030   5.569   0.593   0.988   0.000   0.012
E N  5C6TA                434    0.267  39.045   0.381   0.988   0.000   0.012
E R  5C6TA                435    0.482 110.424   1.718   0.988   0.000   0.012
B A  5C6TA                436    0.056   6.204   0.525   0.988   0.000   0.012
B L  5C6TA                437    0.048   8.734   0.391   0.988   0.000   0.012
E A  5C6TA                438    0.261  28.762   0.328   0.988   0.000   0.012
E Q  5C6TA                439    0.332  59.259   0.661   0.988   0.000   0.012
B I  5C6TA                440    0.040   7.492   0.705   0.988   0.000   0.012
B A  5C6TA                441    0.040   4.364  -0.092   0.988   0.000   0.012
E E  5C6TA                442    0.392  68.430   0.923   0.988   0.000   0.012
B A  5C6TA                443    0.072   7.945  -0.197   0.970   0.001   0.030
B W  5C6TA                444    0.056  13.516   0.812   0.970   0.001   0.030
B C  5C6TA                445    0.043   6.065   0.518   0.923   0.002   0.076
E V  5C6TA                446    0.380  58.452   1.483   0.858   0.002   0.139
B D  5C6TA                447    0.222  32.048  -0.046   0.782   0.003   0.216
B Q  5C6TA                448    0.160  28.612   0.611   0.782   0.003   0.216
E R  5C6TA                449    0.382  87.386  -0.023   0.694   0.003   0.303
E R  5C6TA                450    0.320  73.211   0.299   0.858   0.002   0.139
B T  5C6TA                451    0.206  28.600  -0.541   0.938   0.007   0.055
B L  5C6TA                452    0.171  31.365   0.536   0.938   0.007   0.055
E E  5C6TA                453    0.244  42.627   0.850   0.970   0.001   0.030
B V  5C6TA                454    0.062   9.591   0.682   0.970   0.001   0.030
B F  5C6TA                455    0.056  11.159   0.773   0.970   0.001   0.030
E K  5C6TA                456    0.338  69.588   0.431   0.970   0.001   0.030
B E  5C6TA                457    0.228  39.744  -0.732   0.923   0.002   0.076
B L  5C6TA                458    0.069  12.689  -0.246   0.858   0.002   0.139
E S  5C6TA                459    0.279  32.711   0.143   0.694   0.003   0.303
E K  5C6TA                460    0.583 119.841   0.672   0.339   0.016   0.645
B I  5C6TA                461    0.100  18.537  -0.183   0.115   0.016   0.868
E N  5C6TA                462    0.284  41.622  -0.176   0.016   0.005   0.979
B P  5C6TA                463    0.161  22.846  -1.302   0.502   0.002   0.495
E S  5C6TA                464    0.308  36.074  -0.947   0.782   0.003   0.216
B A  5C6TA                465    0.200  22.051  -0.402   0.879   0.010   0.111
B I  5C6TA                466    0.074  13.671  -0.714   0.879   0.010   0.111
B L  5C6TA                467    0.059  10.821   0.540   0.938   0.007   0.055
B S  5C6TA                468    0.194  22.678  -0.886   0.879   0.010   0.111
B A  5C6TA                469    0.186  20.497  -0.857   0.879   0.010   0.111
B I  5C6TA                470    0.111  20.627  -0.841   0.802   0.014   0.185
B Y  5C6TA                471    0.195  41.629  -0.069   0.622   0.015   0.363
E N  5C6TA                472    0.591  86.522  -0.822   0.181   0.016   0.803
E K  5C6TA                473    0.340  69.856   0.704   0.181   0.016   0.803
E P  5C6TA                474    0.395  56.008  -0.290   0.502   0.102   0.396
B I  5C6TA                475    0.121  22.459   0.253   0.502   0.102   0.396
B A  5C6TA                476    0.138  15.197  -0.309   0.538   0.173   0.289
B A  5C6TA                477    0.123  13.577  -0.545   0.605   0.105   0.290
E R  5C6TA                478    0.362  82.921   0.597   0.605   0.105   0.290
B F  5C6TA                479    0.252  50.556   0.221   0.502   0.102   0.396
B M  5C6TA                480    0.149  29.915  -0.066   0.386   0.097   0.517
B G  5C6TA                481    0.213  16.755  -1.275   0.191   0.086   0.723
B D  5C6TA                482    0.182  26.241  -1.138   0.118   0.150   0.732
B V  5C6TA                483    0.049   7.485   0.209   0.079   0.592   0.329
B L  5C6TA                484    0.056  10.199   0.642   0.085   0.775   0.140
B G  5C6TA                485    0.071   5.588   0.142   0.072   0.855   0.073
B L  5C6TA                486    0.050   9.137   0.207   0.087   0.683   0.230
B A  5C6TA                487    0.144  15.891  -0.670   0.074   0.484   0.442
E S  5C6TA                488    0.455  53.326   1.147   0.022   0.359   0.619
B C  5C6TA                489    0.088  12.411  -0.043   0.022   0.552   0.426
B V  5C6TA                490    0.210  32.292   0.679   0.021   0.756   0.223
E T  5C6TA                491    0.642  89.115   1.077   0.023   0.655   0.322
B I  5C6TA                492    0.117  21.589   1.082   0.021   0.451   0.528
E N  5C6TA                493    0.520  76.113   0.541   0.004   0.085   0.910
E Q  5C6TA                494    0.453  80.906   0.143   0.018   0.047   0.935
E T  5C6TA                495    0.663  92.000  -0.593   0.056   0.142   0.802
E S  5C6TA                496    0.301  35.277   0.419   0.021   0.451   0.528
B V  5C6TA                497    0.077  11.804  -0.050   0.018   0.846   0.136
E K  5C6TA                498    0.329  67.716   1.665   0.011   0.918   0.071
B V  5C6TA                499    0.105  16.123  -0.144   0.011   0.918   0.071
B L  5C6TA                500    0.202  36.950   0.240   0.018   0.846   0.136
E R  5C6TA                501    0.370  84.753  -0.419   0.023   0.655   0.322
B D  5C6TA                502    0.225  32.394  -1.577   0.021   0.451   0.528
B M  5C6TA                503    0.102  20.390  -0.590   0.023   0.655   0.322
E N  5C6TA                504    0.326  47.785  -0.347   0.023   0.655   0.322
B V  5C6TA                505    0.180  27.681   0.204   0.022   0.359   0.619
E K  5C6TA                506    0.481  98.983  -0.451   0.004   0.138   0.858
E E  5C6TA                507    0.769 134.274  -0.606   0.005   0.015   0.979
E S  5C6TA                508    0.604  70.824  -0.174   0.003   0.003   0.994
E P  5C6TA                509    0.691  98.053  -0.329   0.003   0.003   0.994
E G  5C6TA                510    0.561  44.190  -0.125   0.005   0.045   0.951
E R  5C6TA                511    0.230  52.762   1.226   0.005   0.262   0.733
B C  5C6TA                512    0.032   4.507  -0.574   0.004   0.616   0.381
B Y  5C6TA                513    0.130  27.738   0.377   0.003   0.718   0.279
B S  5C6TA                514    0.114  13.314  -2.023   0.004   0.514   0.481
B R  5C6TA                515    0.252  57.616   0.641   0.005   0.262   0.733
B P  5C6TA                516    0.107  15.212  -0.290   0.004   0.085   0.910
B V  5C6TA                517    0.219  33.599   0.176   0.005   0.336   0.660
B V  5C6TA                518    0.052   7.992  -0.318   0.001   0.900   0.099
B I  5C6TA                519    0.116  21.497  -0.247   0.001   0.959   0.040
B F  5C6TA                520    0.047   9.413   0.566   0.001   0.959   0.040
E N  5C6TA                521    0.311  45.530   0.791   0.001   0.959   0.040
B F  5C6TA                522    0.110  22.117   0.271   0.001   0.900   0.099
E A  5C6TA                523    0.305  33.666  -0.196   0.004   0.514   0.481
E N  5C6TA                524    0.356  52.162  -0.996   0.005   0.262   0.733
E S  5C6TA                525    0.596  69.804  -1.423   0.018   0.047   0.935
E S  5C6TA                526    0.540  63.311  -1.600   0.018   0.088   0.893
E Y  5C6TA                527    0.540 115.419   0.246   0.022   0.552   0.426
E V  5C6TA                528    0.335  51.536   0.043   0.021   0.756   0.223
B Q  5C6TA                529    0.232  41.507   0.511   0.018   0.846   0.136
E Y  5C6TA                530    0.488 104.371   0.642   0.021   0.756   0.223
B G  5C6TA                531    0.087   6.871  -2.630   0.022   0.552   0.426
E Q  5C6TA                532    0.338  60.420  -0.233   0.022   0.359   0.619
B L  5C6TA                533    0.149  27.282  -0.336   0.020   0.205   0.775
B G  5C6TA                534    0.199  15.685  -0.277   0.018   0.088   0.893
E E  5C6TA                535    0.572  99.876   0.166   0.052   0.084   0.864
E D  5C6TA                536    0.516  74.298  -0.227   0.053   0.043   0.903
B N  5C6TA                537    0.146  21.374  -0.274   0.052   0.084   0.864
B E  5C6TA                538    0.181  31.586  -0.479   0.069   0.386   0.545
B I  5C6TA                539    0.087  16.169   0.082   0.079   0.592   0.329
B L  5C6TA                540    0.143  26.110   1.288   0.079   0.592   0.329
B L  5C6TA                541    0.132  24.133   0.641   0.069   0.386   0.545
B G  5C6TA                542    0.279  21.934  -0.739   0.056   0.142   0.802
E N  5C6TA                543    0.429  62.806  -0.083   0.052   0.084   0.864
E H  5C6TA                544    0.561 101.991  -0.591   0.052   0.084   0.864
B R  5C6TA                545    0.235  53.792   0.732   0.066   0.296   0.638
E T  5C6TA                546    0.313  43.455  -0.037   0.074   0.484   0.442
E E  5C6TA                547    0.275  48.130   0.576   0.074   0.484   0.442
E E  5C6TA                548    0.546  95.439   1.308   0.148   0.418   0.435
B C  5C6TA                549    0.061   8.564   0.109   0.056   0.142   0.802
E Q  5C6TA                550    0.580 103.570   0.832   0.052   0.084   0.864
E L  5C6TA                551    0.603 110.428   0.462   0.053   0.043   0.903
E P  5C6TA                552    0.629  89.298   0.084   0.018   0.019   0.964
B S  5C6TA                553    0.175  20.510  -0.005   0.018   0.088   0.893
E L  5C6TA                554    0.419  76.682   1.082   0.003   0.718   0.279
B K  5C6TA                555    0.232  47.743   1.708   0.001   0.900   0.099
B I  5C6TA                556    0.069  12.802   1.261   0.001   0.959   0.040
B F  5C6TA                557    0.045   8.951   0.836   0.001   0.959   0.040
B I  5C6TA                558    0.142  26.362   0.379   0.001   0.959   0.040
B A  5C6TA                559    0.064   7.086  -0.240   0.001   0.900   0.099
E G  5C6TA                560    0.332  26.128  -0.178   0.004   0.197   0.799
E N  5C6TA                561    0.553  80.988  -1.148   0.005   0.015   0.979
E S  5C6TA                562    0.331  38.746  -0.692   0.004   0.514   0.481
B A  5C6TA                563    0.124  13.665  -0.114   0.001   0.900   0.099
B Y  5C6TA                564    0.121  25.943   0.947   0.001   0.959   0.040
B E  5C6TA                565    0.209  36.600   1.144   0.001   0.959   0.040
B Y  5C6TA                566    0.106  22.588   0.519   0.001   0.959   0.040
E V  5C6TA                567    0.345  53.011   0.748   0.002   0.816   0.182
B D  5C6TA                568    0.335  48.288  -0.574   0.004   0.514   0.481
B Y  5C6TA                569    0.203  43.488   0.967   0.004   0.514   0.481
E L  5C6TA                570    0.436  79.740   0.458   0.002   0.816   0.182
B F  5C6TA                571    0.207  41.605   0.660   0.002   0.816   0.182
E K  5C6TA                572    0.420  86.394   0.598   0.018   0.846   0.136
E R  5C6TA                573    0.382  87.592   0.405   0.021   0.756   0.223
E M  5C6TA                574    0.514 102.811   0.338   0.022   0.552   0.426
B I  5C6TA                575    0.121  22.385   0.894   0.004   0.420   0.576
E D  5C6TA                576    0.533  76.849   0.935   0.005   0.262   0.733
B L  5C6TA                577    0.129  23.657  -0.150   0.018   0.088   0.893
E S  5C6TA                578    0.662  77.551  -0.439   0.053   0.043   0.903
E S  5C6TA                579    0.531  62.210   0.747   0.113   0.043   0.844
B I  5C6TA                580    0.097  17.908   0.841   0.307   0.165   0.527
E S  5C6TA                581    0.483  56.561  -0.006   0.321   0.252   0.427
E T  5C6TA                582    0.270  37.449   0.706   0.352   0.332   0.316
B V  5C6TA                583    0.070  10.836   0.782   0.478   0.309   0.214
E D  5C6TA                584    0.340  49.008  -0.276   0.578   0.229   0.194
B S  5C6TA                585    0.164  19.197  -0.095   0.649   0.163   0.188
B M  5C6TA                586    0.120  24.092   0.698   0.649   0.163   0.188
B I  5C6TA                587    0.136  25.141   0.944   0.649   0.163   0.188
E A  5C6TA                588    0.441  48.642   0.103   0.578   0.229   0.194
B L  5C6TA                589    0.111  20.287   0.768   0.231   0.330   0.439
E D  5C6TA                590    0.391  56.372  -0.219   0.064   0.216   0.721
B I  5C6TA                591    0.077  14.153   0.445   0.020   0.205   0.775
E D  5C6TA                592    0.428  61.732   0.305   0.069   0.386   0.545
B P  5C6TA                593    0.215  30.494   0.622   0.069   0.386   0.545
B L  5C6TA                594    0.113  20.654   0.336   0.064   0.216   0.721
E E  5C6TA                595    0.446  78.004  -0.079   0.052   0.084   0.864
E N  5C6TA                596    0.471  68.881  -1.246   0.053   0.043   0.903
B T  5C6TA                597    0.216  29.959   1.027   0.018   0.088   0.893
E D  5C6TA                598    0.476  68.592   0.067   0.018   0.088   0.893
B F  5C6TA                599    0.098  19.769   0.907   0.066   0.296   0.638
E R  5C6TA                600    0.378  86.585   1.368   0.079   0.592   0.329
B V  5C6TA                601    0.216  33.184   0.822   0.085   0.775   0.140
B L  5C6TA                602    0.104  19.079   0.882   0.085   0.775   0.140
E E  5C6TA                603    0.331  57.843  -0.056   0.085   0.775   0.140
B L  5C6TA                604    0.116  21.295   0.300   0.079   0.592   0.329
B Y  5C6TA                605    0.168  35.816   0.677   0.148   0.418   0.435
B S  5C6TA                606    0.227  26.569   0.377   0.199   0.152   0.649
E Q  5C6TA                607    0.523  93.354   0.702   0.660   0.049   0.291
E K  5C6TA                608    0.558 114.842   0.102   0.622   0.015   0.363
B E  5C6TA                609    0.200  34.992  -0.204   0.751   0.050   0.199
B L  5C6TA                610    0.254  46.434   0.099   0.751   0.050   0.199
E R  5C6TA                611    0.490 112.118   0.216   0.622   0.015   0.363
E S  5C6TA                612    0.458  53.654  -0.725   0.522   0.016   0.462
B S  5C6TA                613    0.270  31.597  -0.463   0.339   0.016   0.645
E N  5C6TA                614    0.425  62.161  -0.907   0.113   0.043   0.844
B V  5C6TA                615    0.156  24.008   0.015   0.184   0.043   0.773
B F  5C6TA                616    0.160  32.212   0.541   0.184   0.043   0.773
E D  5C6TA                617    0.338  48.677   0.738   0.184   0.043   0.773
B L  5C6TA                618    0.120  21.899   0.795   0.858   0.002   0.139
E E  5C6TA                619    0.375  65.478   0.185   0.923   0.002   0.076
E E  5C6TA                620    0.329  57.459   0.618   0.923   0.002   0.076
B I  5C6TA                621    0.055  10.194   0.518   0.923   0.002   0.076
B M  5C6TA                622    0.119  23.892   0.537   0.938   0.007   0.055
E R  5C6TA                623    0.355  81.341  -0.229   0.879   0.010   0.111
B E  5C6TA                624    0.272  47.466  -0.427   0.802   0.014   0.185
B F  5C6TA                625    0.167  33.477  -0.062   0.717   0.014   0.269
B N  5C6TA                626    0.242  35.414  -0.680   0.622   0.015   0.363
B S  5C6TA                627    0.206  24.096  -0.447   0.717   0.014   0.269
B Y  5C6TA                628    0.225  48.104  -0.222   0.802   0.014   0.185
E K  5C6TA                629    0.304  62.553   0.593   0.802   0.014   0.185
E Q  5C6TA                630    0.356  63.492   0.429   0.802   0.014   0.185
E R  5C6TA                631    0.377  86.379   0.646   0.802   0.014   0.185
B V  5C6TA                632    0.141  21.626  -0.628   0.622   0.015   0.363
E K  5C6TA                633    0.492 101.122   0.717   0.430   0.016   0.555
E Y  5C6TA                634    0.623 133.199   0.322   0.181   0.016   0.803
B V  5C6TA                635    0.200  30.771  -0.253   0.115   0.016   0.868
E E  5C6TA                636    0.909 158.820   0.962   0.003   0.003   0.994

Explain the output.  Go back.
```

---
